# Supplementary figures and images for: Molecular characterization and population genetics of Theileria parva in Burundi’s unvaccinated cattle: Towards the introduction of East Coast fever vaccine
Source: PLoS One. 2021 May 17;16(5):e0251500. doi: 10.1371/journal.pone.0251500 (PMC8128232; doi:10.1371/journal.pone.0251500)

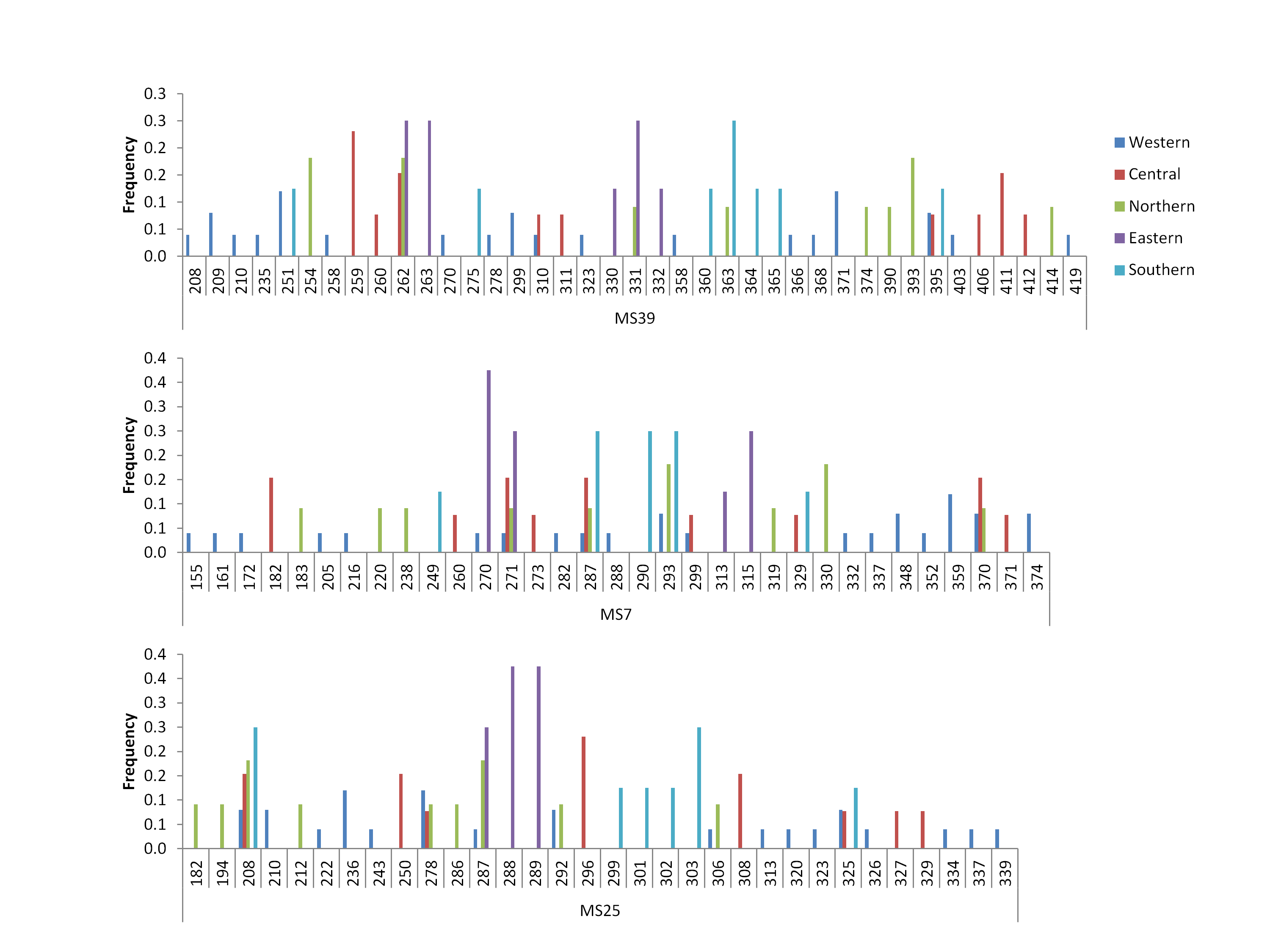

Supplement: S1 Fig — The allele frequencies from Burundi field samples in this study showing the presence of both shared and unique alleles. MS39 shows the highest number of unique alleles while MS19 shows the least. A total of 4 shared alleles is observed on each loci. (TIF) [file pone.0251500.s001.tif]

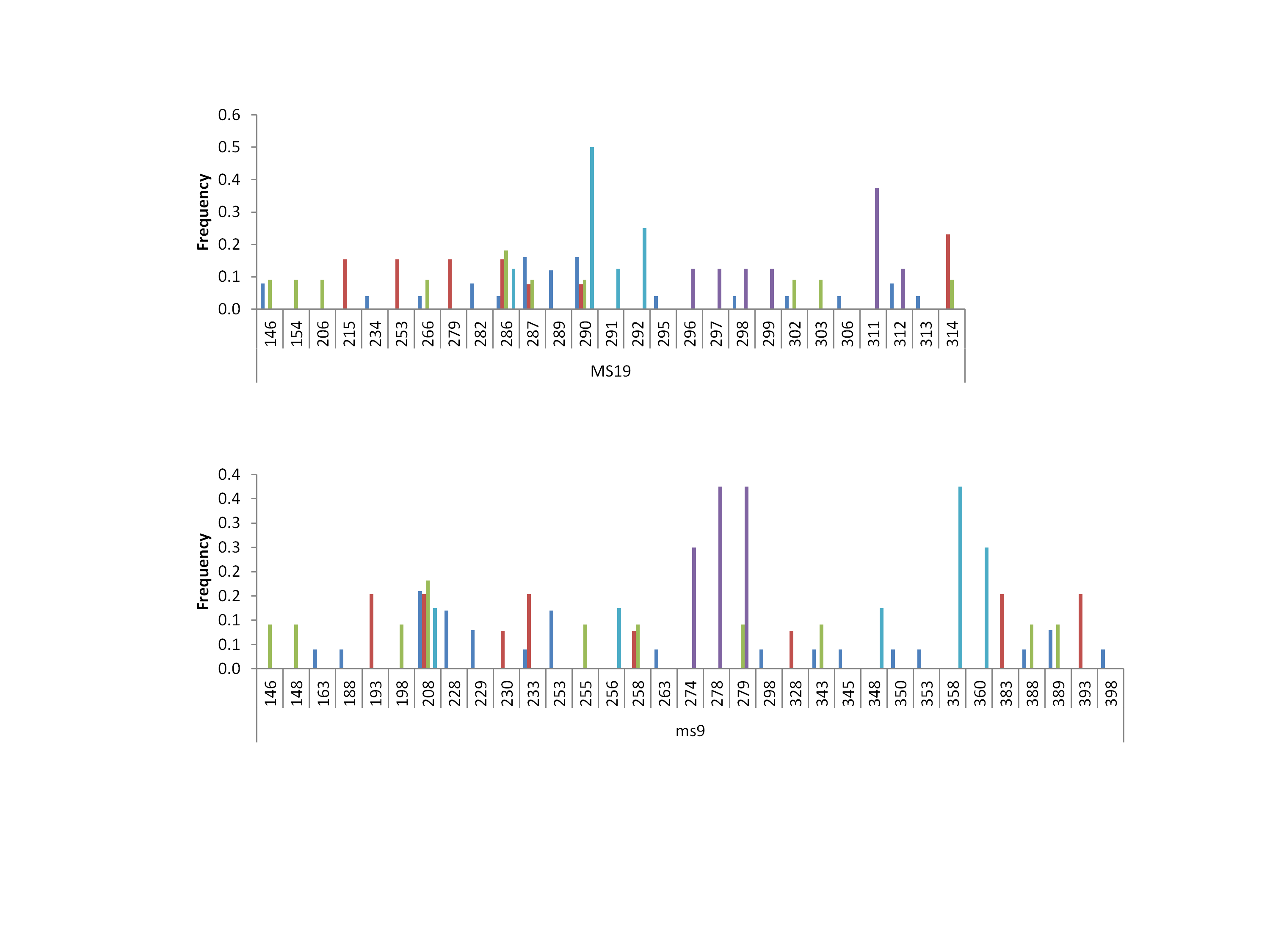

Supplement: S2 Fig — The allele frequencies from Burundi field samples in this study showing the presence of both shared and unique alleles. MS39 shows the highest number of unique alleles while MS19 shows the least. A total of 4 shared alleles is observed on each loci. (TIF) [file pone.0251500.s002.tif]

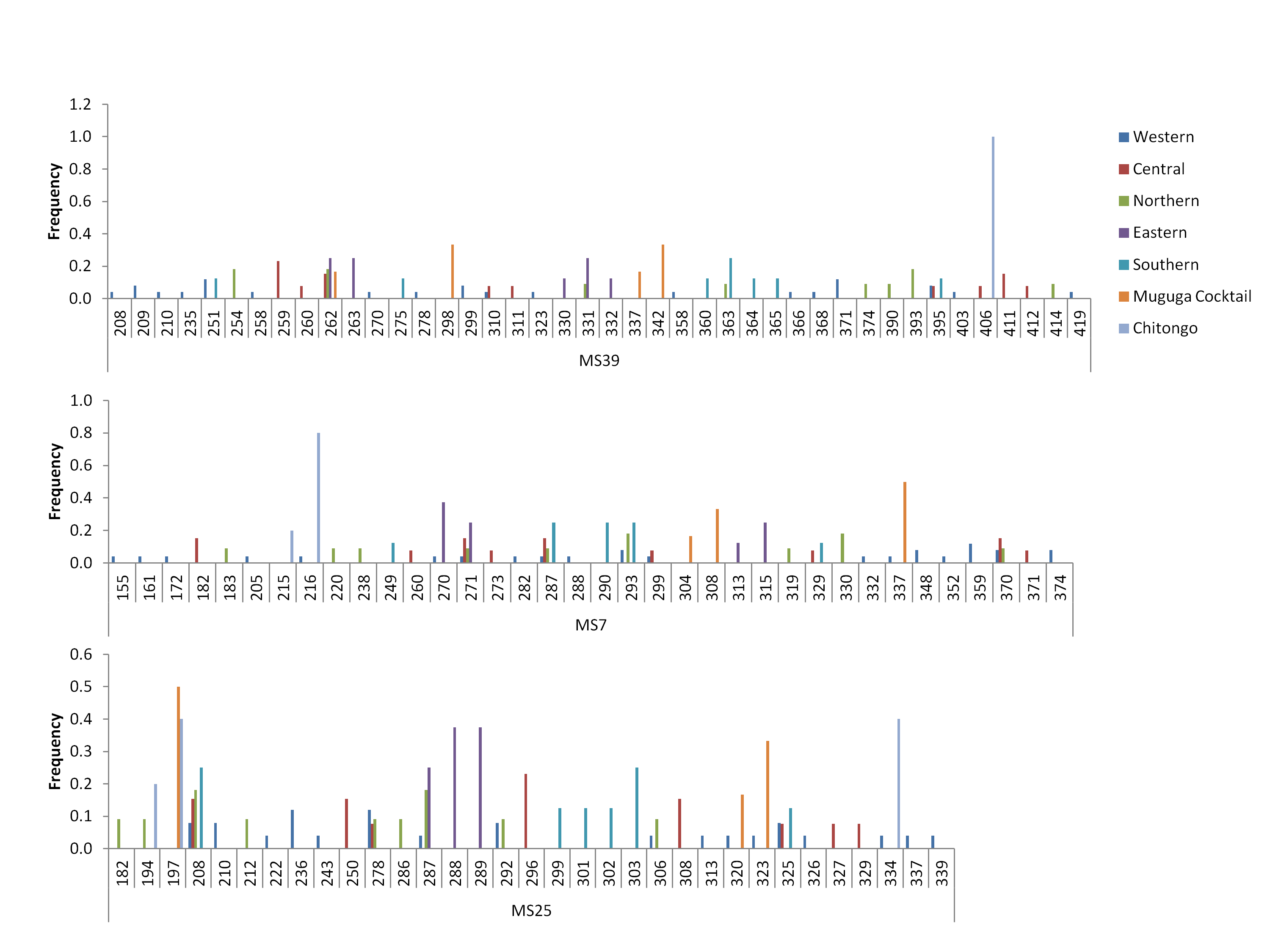

Supplement: S3 Fig — Muguga cocktail shares only two alleles with Burundi field samples on loci MS19 and ms9 while none are shared on the remaining loci. Chitongo vaccine stock does not share any alleles with the Burundi field samples. (TIF) [file pone.0251500.s003.tif]

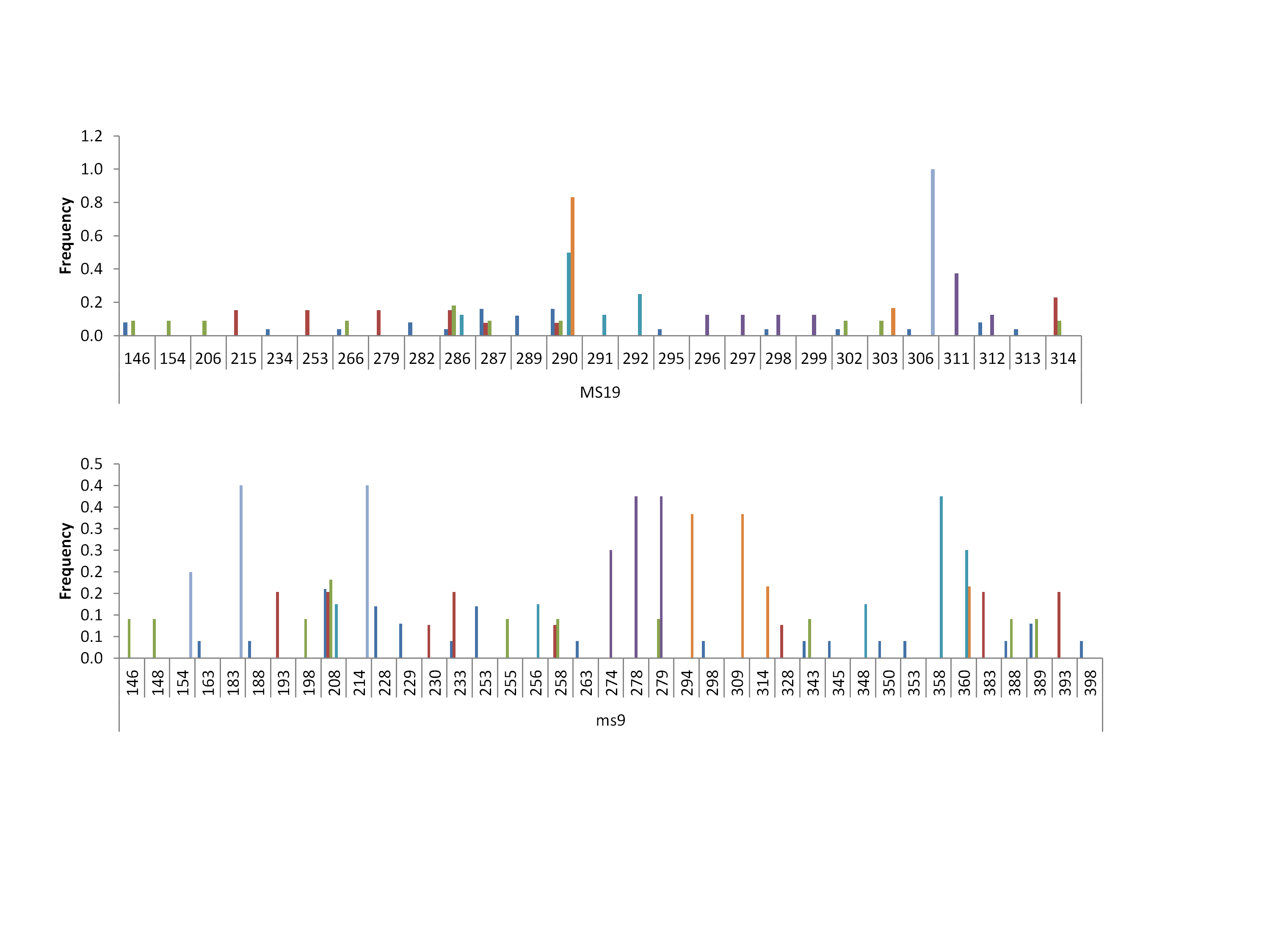

Supplement: S4 Fig — Muguga cocktail shares only two alleles with Burundi field samples on loci MS19 and ms9 while none are shared on the remaining loci. Chitongo vaccine stock does not share any alleles with the Burundi field samples. (TIF) [file pone.0251500.s004.tif]
