# Supplementary material for: Molecular characterization and population genetics of Theileria parva in Burundi’s unvaccinated cattle: Towards the introduction of East Coast fever vaccine
Source: PLoS One. 2021 May 17;16(5):e0251500. doi: 10.1371/journal.pone.0251500 (PMC8128232; doi:10.1371/journal.pone.0251500)
Supplement: S1 Table — (DOCX) [file pone.0251500.s005.docx]

**S1 Table**. Theileria parva satellite markers used in this study to genotype Burundi field samples, Muguga cocktail and Chitongo vaccines stocks.

| **Marker** | **Chromosome Number** | **Size (bp)** | **Reference** |
| --- | --- | --- | --- |
| MS7 | 1 | 372 | Patel et al., 2011 |
| MS19 | 2 | 307 | Patel et al., 2011 |
| MS25 | 3 | 325 | Patel et al., 2011 |
| ms9 | 3 | 230 | Patel et al., 2011 |
| MS39 | 4 | 263 | Patel et al., 2011 |
