# Supplementary material for: Molecular characterization and population genetics of Theileria parva in Burundi’s unvaccinated cattle: Towards the introduction of East Coast fever vaccine
Source: PLoS One. 2021 May 17;16(5):e0251500. doi: 10.1371/journal.pone.0251500 (PMC8128232; doi:10.1371/journal.pone.0251500)
Supplement: S2 Table — (DOCX) [file pone.0251500.s006.docx]

| **Gene** | **Group number** | **Representative Sample** | **Number of Samples** | **Samples** |
| --- | --- | --- | --- | --- |
| **Tp1** | 1 | Buba07B52 | 5 | Mar37B57, Rubi37B61, Ruta40B90 |
|  | 2 | Buba22B50 | 24 | Buba62B45, Buba69, Buba101, Gae2, Gae52, GaeE54, Gat51, GatH67, Gite44, Kaya02, Kaya13, Kiru18, Kiru19, Kiru22, Kiru34, Kiru39, Kiru59, Kiru73,MurC14, Rubi24, Rubi54, Ruta5, Ruta11 |
|  | 3 | Buba48 | 16 | Buba53, Buba60, Gae15, Kaya26, Kaya27, Kaya31, Kaya35, Kiru49, Mar4, MurE63, MurE73, MWE36, MwE66, MwE77, Ruta23, |
|  | 4 | Buba58 | 15 | Buba96, Buke19, GaE29, GatM45, Gite505, Gite555, Kaya20, Kiru16, Kiru76, Kiru81, Mur3, MwE45, Rubi19, Ruta55 |
|  | 5 | Buba75 | 3 | Buba97, GatK18, |
|  | 6 | Buba76 | 3 | GatS10, Ruta30 |
|  | 7 | GatS15 | 2 | Mar65 |
|  | 8 | Kaya16 | 5 | Kiru12, Kiru51, Mugo51, MvA100 |
|  | 9 | Kiru24 | 4 | Mar81, Ruta15, Ruta54, |
|  | 10 | Kiru46 | 2 | Ruta53 |
|  | 11 | Mugo30 | 2 | Rubi48 |
|  | 12 | Mur68 | 2 | Ruta33 |
|  |  |  |  |  |
| **Tp2** | 1 | Buba20 | 4 | Kaya93, Rubi44, Ruta54, |
|  | 2 | Buba22 | 31 | Buba49, Buba62, Buba76, GaM45, GatS11, GatS15, Gite538, Kaya12, Kaya17, Kaya26, Kaya27, Kaya31, Kiru22, Kiru34, Kiru49, Kiru73, Kiru76, Kiru81, MarS49, Mugo30, MugoC87, MurE63, MwE36, Rubi52, Ruta24, Ruta41, Ruta56, Ruta78, Ruta80, Ruta98 |
|  | 3 | Buba53 | 5 | Canku10, Kaya02, MurE40, Ruta23 |
|  | 4 | GaE112 | 5 | Kiru77, Mar76, Mugo48, Rubi37 |
|  | 5 | GaS14 | 6 | Kaya20, Mar81, MurC14, MwA55, Ruta55 |

**S2 Table.** Summarized list of field sample sequences with 100% nucleotide homology used for the generation of phylogenetic trees for the Tp1 and Tp2 genes.
